# Association of the Unstimulated Whole Salivary Cytokine IL-1β Levels with Initial, Moderate and Severe Periodontitis. A Case Control Study

**DOI:** 10.3390/ijerph19052889

**Published:** 2022-03-02

**Authors:** Muhammad M. Majeed, Imtiaz Ahmed, Talat Roome, Yasser Alali, Khulud A. Al-Aali, Naseer Ahmed, Zohra Saleem, Abdulkareem A. Alhumaidan, Waqas A. Farooqui, Saeeda Ahmed, Fahim Vohra, Tariq Abduljabbar

**Affiliations:** 1 Department of Oral Biology, Altamash Institute of Dental Medicine, Karachi 75500, Pakistan; mmansoormajeed@gmail.com; 2 Department of Orthodontics, Dow University of Health Sciences, Karachi 74200, Pakistan; imtiaz.ahmed@duhs.edu.pk; 3 Section of Molecular Pathology, Dow University of Health Sciences, Karachi 74200, Pakistan; talat.roome@duhs.edu.pk; 4 Department of Maxillofacial Surgery, College of Dentistry, King Saud University, Riyadh 11451, Saudi Arabia; yalali@ksu.edu.sa; 5 Department of Clinical Dental Sciences, College of Dentistry, Princess Nourah Bint Abdulrahman University, Riyadh 11564, Saudi Arabia; kaalaali@pnu.edu.sa; 6 Department of Prosthodontics, Altamash Institute of Dental Medicine, Karachi 75500, Pakistan; naprosthodontist@gmail.com; 7 Deprtment of Oral and Maxillofacial Surgery, Dow University of Health Sciences, Karachi 74200, Pakistan; zohra_mymani@hotmail.co.uk; 8 Preventive Dental Sciences Department, College of Dentistry, Imam Abdulrahman bin Faisal University, Dammam 34212, Saudi Arabia; aaalhumaidan@iau.edu.sa; 9 School of Public Health, Dow University of Health Sciences, Karachi 74200, Pakistan; waqas.ahmed@duhs.edu.pk; 10 Department of Public Health, Baqai Mecial University, Karachi 75340, Pakistan; dr.saeeda.bashir@hotmail.com; 11 Department of Prosthetic Dental Sciences, College of Dentistry, King Saud University, Riyadh 12372, Saudi Arabia; fvohra@ksu.edu.sa

**Keywords:** periodontitis, initial, moderate, severe, interleukin, inflammation, disease

## Abstract

Periodontitis (P) is a highly prevalent inflammatory disease of the oral cavity. The objective of the study was to evaluate the stages of pro-inflammatory cytokine IL-1β in initial, moderate and severe periodontitis. One hundred and twenty two patients were included in the study. Periodontitis subjects had at least 20 natural teeth and ≥8 sites with pocket depths of >4 mm and clinical attachment loss (CAL). A questionnaire was used with respect to the socio demographic parameters which included age, gender, ethnicity, education, marital, residence and occupation. To categorize the severity of the disease, teeth were assessed for, Plaque index (PI), Bleeding on probing (BOP), CAL, missing tooth, tooth mobility and bone loss. Unstimulated whole saliva (UWS) was collected and Interleukin-1β (IL-1β) cytokine levels were analyzed using enzyme linked immunosorbent assay with microplate reader at 450 nm. Clinical parameters and salivary cytokine concentrations were assessed using one-way analysis of variance, whereas a correlation of cases with gender and severity of periodontitis was evaluated using chi-square test. Fifty-nine patients were healthy controls and 63 were periodontitis patients Thirty two percent (*n* = 20) had initial periodontitis, 40% (*n* = 25) suffered from moderate and 29% (*n* = 18) had severe periodontitis. Periodontitis subgroups were significantly different with regards to age and gender (*p* < 0.001). The mean PPD and CAL among the periodontitis patients (PPD, 3.52 ± 1.25 mm; CAL, 4.04 ± 1.64 mm) were significantly compromised (*p* < 0.05) compared to healthy controls (PPD, 1.52 ± 0.73 mm; CAL, 0.08 ± 0.28 mm). Increased levels of IL-1β were associated with high CAL and PPD findings. UWS IL-1β levels were higher in periodontitis patients compared to healthy individuals. In addition, cases of severe periodontitis showed significantly higher UWS IL-1β levels compared to initial and moderate periodontitis patients. Comparative levels of salivary IL-1β can be potentially used as a diagnostic tool for periodontitis identification and disease progression along with clinical parameters.

## 1. Introduction

Infectious bacteria residing in dental plaque are the main initiators for periodontal destruction and disease pathogenesis. Periodontitis is a pathological condition that results in the inflammation and destruction of the tooth-supporting structure known as periodontium over a period [1,2]. The Global Burden of Disease (GBD) Study and other epidemiological studies have reported that 50% of the population suffers from initial-to-moderate periodontitis and 10% face severe forms of the disease. Therefore, it is considered the sixth most prevalent condition [2]. Periodontal disease inflammation is a misbalance of destruction due to reactive oxygen species and antioxidant capacity of periodontal tissues [3,4]. Cytokines and chemokines are pivotal in creating a pro- and anti-inflammatory equilibrium, however if secreted in abundance, these can induce periodontal tissue pathology [5]. The most critical step is the apical migration of the junctional epithelium and the gingival pocket formation, resulting in the accumulation of cells responsible for inflammatory reaction in the gingival tissues [6]. These cells may lead to inflammatory lesions and damaged periodontal tissue attachment and alveolar bone loss [6]. Henceforth, the severity of the periodontal infection raises the risk of a complex multiphase disease of the organ system such as cardiovascular system, endocrine system, reproductive system and the respiratory system

The severity of periodontitis is defined as the destruction of the tooth attachment apparatus leading to tooth loss as the disease progress over a period. Therefore, many consider the diseases as non-resolving chronic inflammation. In the last three decades the definition of periodontitis has evolved due to the availability of new evidence [7]. The recent system of classification combines chronic and aggressive periodontitis as “periodontitis”, further divided into grades and stages [7]. Authors have identified that immediate treatment at initial to moderate periodontitis stage where minimal clinical attachment loss and bleeding on probing is observed, has led to good clinical prognosis [8,9]. Nonetheless, prolonged frequent inflammation leads to low degree systemic inflammation and increased levels of cytokines, such as tumour necrosis factor-α (TNF-α), Interleukin (IL)-1β, IL-4, IL-6 and IL-10 that damages the bone and other supporting structures [10].

Various studies have suggested cytokines’ role in the development and progression of periodontal disease [11,12]. The plaque bacteria trigger a burst of cytokine release that aggravates periodontal destruction. Despite their protective role, these cytokines are involved in various physiologic processes and abnormal chemical change that may induce different pathologies [13,14]. IL-1β is a potent stimulator of osteoclastic activity that indirectly affects the severity of periodontitis in alveolar bone loss [15,16,17]. IL-1β cytokine has been linked with inflammatory tissue damage that plays a vital role in the defense mechanisms against infective and injurious agents. Many cells in the human body produce IL-1β, including monocytes, macrophages and dendritic cells and multiple studies have associated it with periodontitis, alveolar bone loss and rheumatoid arthritis [18,19].

Studies have shown a direct association of periodontitis to the levels of cytokines detected in the saliva [20,21,22]. Interleukin-1β was identified as a potent inducer of bone resorption and connective tissue degradation with increased severity of disease [23]. Gumus et al., proved that bone destruction and inflammation of periodontal ligament and other tissues linked with periodontium may result in a high IL-1β cytokine level [16]. However, does increasing levels of IL-1β as a predictive and therapeutic biomarker, associate with increased severity of clinical periodontitis is not known. It is hypothesized that the increasing salivary IL-1β levels will associate with increasing severity of periodontitis. Therefore, the present study aimed to evaluate the unstimulated whole salivary cytokine levels of pro-inflammatory cytokine IL-1β in periodontitis patients with different severity levels.

## 2. Materials and Methods

### 2.1. Ethical Consideration

This case-control study was performed at Dow University of Health sciences after getting ethical approval from the Institutional Review Board (REF: IRB-758/DUHS/Approval/2016/277) under the standards of the Helsinki declaration (1964). After the approval from the review board, the informed consent was taken from each participant with the right to withdraw from the study at any moment without any consequences. The collected information was confidential and anonymous to reserve the rights of the voluntary participants.

### 2.2. Study Participants 

The current study was conducted at Dow University of Health Sciences, Karachi Pakistan. The study assessed the oral health condition of 122 patients (63 periodonitis patients and 59 healthy controls). The sample size was calculated using the Fleiss method with continuity correction, where *p*-value was set at <0.05 for significance, to have a 95% confidence interval and 80% power. The required sample size obtained through calculations was 20.

The periodontally healthy subjects (controls) were categorized according to 18 to 65 years of age, with at least 20 natural teeth and no pocket depth or clinical attachment level measurements of more than 3 mm, whereas periodontitis (P) was defined as individuals with chronic inflammation of periodontal tissues due to bacterial plaque [3,4]. The periodontitis subjects were 18 to 65 years of age with at least 20 natural teeth and ≥8 sites with pocket depths of more than 4 mm and clinical attachment loss. Periodontitis (P) was staged into initial, moderate and severe, based on clinical attachment loss (CAL) of 1–2 mm CAL (Initial P), 3–4 mm CAL (Mode P) and >5 mm CAL (Severe P) (Table 1). The periodontitis patients were classified in accordance with the classification system as presented in Table 1.

Individuals with orthodontic appliances, abnormal salivary function, use of prescription drugs, use of antibiotics, use of anti-inflammatory medicine, systemic conditions, pregnant, lactating females were excluded. In addition, smokers, gutka, betel quid and tobacco users; and individuals with periodontal therapy within 6 months were also excluded. Participants were enrolled in the study after patients with periodontal disease were observed and screened by a periodontist using the eligibility criteria. Patients were later consented and assigned to study groups.

### 2.3. Questionnaire

A standardized questionnaire was used to gather information with respect to the socio demographic parameters, which included age, gender, ethnicity, education, marital status, residence and occupation. Clinical inflammatory parameters were evaluated, to assess the stages of periodontitis and to demonstrate its association with the increased level of IL-1β. Clinical attachment Loss (CAL), periodontal pocket depth (PPD), bleeding on probing (BOP), plaque index (PI) and missing teeth were evaluated.

### 2.4. Clinical Examination

Clinical periodontal examination was performed by two trained and calibrated investigators (MMM & TR), who were masked to the groups. For inter-examiner reliability, data obtained was crosschecked to reduce internal bias and Kappa scores were calculated. BOP was assessed using the papillary bleeding method including the use of triangular-shaped wooden toothpick to stimulate the interproximal gingival tissue. The index ranged from healthy gingiva, followed by red tissue with no bleeding to copious amount of bleeding. PI was measured using the Loe and Silness Plaque Index on a scale of 0 to 3. For PPD and CAL assessment, a UNC 15 (University of North Carolina) probe was employed at six sites of every tooth except third molars with standard force; attachment loss was the distance from the cemento–enamel junction to the bottom of the pocket [1,15,16]. 

### 2.5. Unstimulated Whole Saliva (UWS) Collection 

Subjects were not allowed to eat or to brush 30 min before sampling. UWS samples were collected in sterile tubes after clinical examination. Samples were collected before probing in order to prevent saliva from blood contamination by passive drooling method. Immediately after the collection, saliva was centrifuged (HERMLE- Z366, Wehingen, Germany) at 3500× *g* for 5 min to remove the debris. Collected saliva was stored at −80 °C until processing. All samples were diluted at a working dilution of 1:100 in phosphate buffered saline.

Identification of Interleukin-1β was attained by using Enzyme linked immunosorbent assay (ELISA) method (SolarBio Science & Technology Co, Ltd., Beijing, China). 10 µL of saliva from each sample was added to designated well of a 96 well plate in replicate, which was already filled with 40 µL of sample diluent provided with the kit. Subsequently, the plate was incubated at 37 °C for 30 min followed by the addition of 10 µL Chromatin Solution A and Chromatin Solution B. The process was completed by adding 50 µL of stop solution to each well. After the storage in dark for 15 min, the microplate was assessed under reader at 450 nm (ELX808, BioTek, Santa Clara, CA, USA). The assay has a sensitivity of less than 0.1 pg/mL minimum detectable dose of cytokines and did not show any cross-reactivity to a wide spectrum of different related cytokines. Data analysis was performed using Statistical program for social sciences (SPSS, IBM, NY, USA). 

### 2.6. Statistical Analysis

The data was computed using the Statistical Program for Social Sciences (SPSS) version 21 (IBM, Armonk, NY, USA). The descriptive statistics (mean and standard deviations) were reported for age, demographic variables and clinical periodontal parameters (PI, BOP, PPD and CAL). Clinical parameters and salivary cytokine concentrations were assessed using one-way analysis of variance (ANOVA) (comparing parameters between periodontitis patients), whereas a correlation of cases with gender and severity of periodontitis was calculated using chi-square test. Comparisons between healthy and periodontitis patients were made using t-test. A *p*-value of 0.05 was considered significant.

## 3. Results 

### 3.1. General Characteristics

One hundred and twenty two subjects were recruited for the study, of which 59 were healthy controls and 63 were periodontitis patients (Table 2). The mean age of healthy and CP patients was 31.44 ± 5.87 years and 33.40 ± 8.28 years respectively. The overall mean age of the participants was 32.5 ± 7.2 years. A significant difference was observed between the healthy, initial, moderate and severe periodontitis patients (*p* < 0.05) (Table 1). Among periodontitis patients, 32% (*n* = 20) had initial periodontitis, 40% (*n* = 25) suffered from moderate and 29% (*n* = 18) had severe periodontitis. These periodontitis subgroups were significantly different with regards to age and gender (*p* < 0.001). 

### 3.2. Clinical Characteristics

The study evaluated clinical inflammatory parameters among healthy and periodontitis patients. The PI and BOP among healthy (PI, 0.74 ± 0.26; BOP, 16.11 ± 5.33) and periodontitis (PI, 2.0 ± 0.48; BOP, 33.70 ± 7.06) patients was significantly different (*p* < 0.05) (Table 3). The mean PPD and CAL among the periodontitis patients (PPD, 3.52 ± 1.25 mm; CAL, 4.04 ± 1.64 mm) were significantly compromised (*p* < 0.05) compared to healthy controls (PPD, 1.52 ± 0.73 mm; CAL, 0.08 ± 0.28 mm). PI, BOP, PPD and CAL, tooth loss and tooth mobility were significantly higher in periodontitis compared to healthy controls (Table 2).

Salivary IL-1β levels among the initial, moderate and severe periodontitis patients are presented in Table 3. IL-1β levels among healthy (0.234 ± 0.299 pg/mL) and periodontitis patients (1.948 ± 1.66 pg/mL) were significantly different (*p* < 0.01) (Table 4). Compared to the healthy patients [0.24 ± 0.30], severe periodontitis [2.89 ± 2.32] presented with the highest cytokine level followed by moderate [1.95 ± 0.99] and mild [0.74 ± 0.83] periodontitis. 20–50% bone loss observed in the severe periodontitis groups can be justified with raised levels of cytokine in the saliva.

The outcomes showed a positive and highly significant correlation between the IL-1β salivary levels and clinical periodontal parameters (PI, BOP, PPD, CAL). The direct relation with clinical parameters and stages of periodontitis measured was PI (0.573), PPD (0.73) and CAL (0.803). Significant levels of plaque are correlated to increased IL-1β leading to a rise in CAL and PPD.

## 4. Discussion

The present study aimed to present the salivary IL-1β concentration and periodontal inflammatory parameters among initial, moderate and severe periodontitis patients. It was observed that salivary IL-1β levels increased and periodontal parameters worsened with increased stages of periodontitis. Therefore the suggested hypothesis was accepted. Microbial toxins have shown to stimulate various inflammatory mediators, including Interleukin-1β and tumor necrosis factor-α. IL-1β levels in gingival crevicular fluid (GCF) of individuals with periodontitis were significantly higher than those in healthy individuals in previous studies [24,25]. A series of events are associated with the high levels of cytokines released, which influences the stages of periodontitis.

Among the recruited participants in the study, significantly different periodontal inflammatory parameters were observed among the periodontitis patients. A study by Sánchez recruited 72 participants, including healthy individuals and cases with initial, moderate and severe forms of periodontitis [25]. They showed significant variation among healthy, moderate and severe periodontitis cases. In the moderate periodontitis group, the concentration of IL-1β usually raised more than five times, whereas, for severe periodontitis groups, it is increased by more than seven times above the level of healthy individuals [19]. However, the difference in IL-1β concentration among healthy individuals and initial periodontitis cases was not significant. In the present study, similar IL-1β concentration was observed in initial and moderate periodontitis, however severe periodontitis showed significantly higher IL-1β levels.

The continuous trigger of the cytokines has shown to slowly deteriorate the surrounding structures, which includes gingival tissue, periodontal ligament and bone. Studies have reported that Interleukin-1β can stimulate the movement of inflammation-causing cells from the blood to the inflamed sites; furthermore, it also signals the extracellular matrix and induces release of other cytokines [19,26]. The present study suggested that PPD and mobility of teeth (indicator of bone loss) was similar in initial and moderate periodontitis, however severe periodontitis showed significantly higher levels of IL-Iβ and periodontal destruction. A study performed in Finland performed in 2009, investigated levels of salivary cytokines to understand periodontitis [27]. Only IL-Iβ was identified in every sample; however, other tested cytokines, TNF-α and IL-6, were also detected in some samples [27]. Thus, in accordance with the findings, the high levels of IL-1β can be linked to the severe form of periodontitis at higher levels. 

It is suggested that toxins present in dental plaque increase inflammatory mediator production, including IL-1β. A study examined the level of IL-Iβ and PTX3 associated with stages of periodontitis. It showed a positive association of both with periodontal disease, which further confirms the findings of our study [28]. In another study, investigating the histo-pathogenesis of inflamed periodontium, revealed a significant increase in leukocyte (neutrophilic granulocytes 60% to 70%) under accumulated plaque with collagen-poor connective tissue at 4 days of infection (initial periodontitis) [29]. However, at 28 days of disease, the infiltrate comprised mainly of mononuclear leukocytes, especially plasma cells; and neutrophils occupied only a small fraction of the infiltrate [16]. This suggests the role of plaque accumulation over a period of time on cellular changes in the tissues and therefore indicates cytokine level changes. In the present study, plaque index was higher in periodontitis patients; and severe form of disease showed higher levels of plaque. Therefore, lack of oral hygiene maintenance and increased plaque accumulation, aggravates the periodontal disease condition leading to irreversible tissue loss.

Apart from high PI, BOP and CAL, the present study observed greater bone loss and tooth mobility as the severity of periodontitis progressed. In recent investigations, osteoclastic bone damage was found to be directly related to the receptor activator of NF-kB ligand (RANKL) produced by osteoblastic cells and periodontal ligament cells. The induced periodontal inflammation triggers essential agents for RANKL induction that regulates osteoclast function and activity [29]. Hence, leading to bone loss in surrounding region and tooth mobility. However, RANKL expression in periodontal tissues is a complicated process that is linked to multiple factors such as age, gender and socio-demographics. Many authors identified a significant correlation between RANKL expression and periodontal pathogenesis, which led to new insights; however, limited data was gathered in accordance with the severity of periodontitis and RANKL expression [29,30]. Future clinical trials investigating the association of RANKL and different stages of periodontitis are recommended. 

Multiple studies have provided supporting evidence related to the different concentrations of IL-1β in saliva, crevicular fluid and serum especially among periodontitis patients [29,30,31]. It was identified that the IL-Iβ mainly acts locally; therefore, serum levels measured were low compared to the crevicular fluid [32]. However, studies have presented comparable differences in cytokine levels in both, saliva and GCF of periodontitis patients [32,33,34]. GCF and saliva are two interconnected systems, where cytokines travel from GCF to saliva in inflammatory response; nonetheless, various cytokines levels present differently according to the cellular response [32]. Furthermore, it was observed that IL-1β was higher in GCF compared to IL-9 levels in moderate periodontitis sites [32]. However, GCF presents challenges in collecting samples from multiple sites, for acquiring a representative specimen, resulting in high probability of tissue damage and increasing specimen collection duration. 

Within certain clinical limitations, the study showed a significant correlative rise in cytokine levels with severity of the periodontal inflammation. As a possible study limitation, it is pertinent to mention that specimen collection was not site specific and UWS was collected which could have resulted in contamination of the sample. Therefore, lower levels of IL-1β maybe due to the dilution or contamination [28,33,34]. Furthermore, the study excluded confounding factors such as smoking and diabetes mellitus; both are known to increase the risk of periodontitis. Moreover, only a pro-inflammatory cytokine IL-1β was assessed in the present study. However the balance of pro and anti-inflammatory cytokines is suggested to influence periodontal disease progression and severity. Therefore further randomized controlled trials investigating the association of pro and anti-inflammatory cytokines in site-specific samples for different stages of periodontitis are recommended. 

## 5. Conclusions

The unstimulated whole salivary (UWS) IL-1β levels were higher in periodontitis patients compared to healthy individuals. In addition, cases of severe periodontitis showed significantly higher UWS IL-1β levels compared to initial and moderate periodontitis patients. Comparative levels of salivary IL-1β can therefore be potentially used as a diagnostic tool for the identification of periodontal disease progression along with clinical parameters. 

## Figures and Tables

**Table 1:** Staging and grading of Periodontitis.

(a) Stages based on severity and complexity of management	Stage I: Initial Periodontitis Stage II: Moderate Periodontitis Stage III: Severe Periodontitis with Potential for Additional Tooth Loss Stage IV: Severe Periodontitis with Potential for Loss of Dentition
(b) Extent and distribution	Localized; generalized; molar-incisor distribution.
(c) Grades	Grade A: Slow rate of progression Grade B: Moderate rate of progression Grade C: Rapid rate of progression

**Table 2:** The Mean and standard deviation for the descriptive parameters for healthy controls and periodontitis patients.

Status	Healthy	Initial Perio	Mod Perio	Severe Perio
Age	31.44 ± 5.87	33.40 ± 8.28	32.10 ± 6.11	34.80 ± 7.84
Male	35 (59.32%)	13 (20.63%)	17 (26.98%)	10 (15.87%)
Female	24 (40.67%)	7 (11.11%)	8 (12.69%)	8 (12.69%)

Perio: Periodontitis.

**Table 3:** The mean and standard deviation of the clinical parameter between periodontitis and healthy controls.

Clinical Parameters	Healthy Controls	Periodontitis	*p*-Value *
PI	0.74 ± 0.26	2.01 ± 0.48	<0.001
BOP	16.11 ± 5.33	33.70 ± 7.06	<0.001
PPD (mm)	1.52 ± 0.73	3.52 ± 1.25	<0.001
CAL (mm)	0.08 ± 0.28	4.04 ± 1.64	<0.001
Tooth Loss	0.33 ± 0.10	1.30 ± 0.52	>0.001
Mobile tooth	0.42 ±0.15	2.62 ± 1.18	<0.001

* *t*-test, for comparing parameters between healthy and periodontitis patients.

**Table 4:** Mean and standard deviation of clinical parameter with respect to the stages of periodontitis.

Periodontitis	CAL (mm)	PPD (mm)	Mobility of Teeth (Score)	IL-1β	*p* Value *
Initial	2.35 mm ± 0.28 ^a^	2.90 mm ± 0.73 ^a^	1.25 ± 0.73 ^a^	0.815 pg/mL ± 0.492 ^a^	*p* < 0.001
Moderate	4.08 mm ± 0.48 ^b^	3.12 mm ± 0.78 ^a^	1.55 ± 0.59 ^a^	2.021 pg/mL ± 0.445 ^a^	
Severe	5.97 mm ± 0.49 ^c^	4.77 mm ± 0.30 ^b^	5.57 ± 0.69 ^b^	3.16 pg/mL ± 0.592 ^c^	
*p* value *	0.021	0.043	0.001	0.013	

* ANOVA, different superscript small alphabets in same column denote statistical significant difference.

## Data Availability

The study data is available on contact from corresponding author.
